# Effect of silver nanoparticles on salt tolerance of *Satureja hortensis* l. during *in vitro* and *in vivo* germination tests

**DOI:** 10.1016/j.heliyon.2021.e05981

**Published:** 2021-02-12

**Authors:** Fatemeh Nejatzadeh

**Affiliations:** Department of Horticulture, Khoy Branch, Islamic Azad University, Khoy, Iran

**Keywords:** Germination rate, Salinity resistance, Nano-silver, (*Satureja hortensis* L)

## Abstract

This study to evaluate the effects of silver nanoparticles on the salinity tolerance of (*Satureja hortensis* L.). The study done based on factorial experiment using a completely randomized design, in a laboratory and greenhouse in Islamic Azad University of Khoy, Iran in 2015. Silver nanoparticles concentrations were 0, 40, 60, and 80 ppm and salt concentrations 0, 30, 60, 90, and 120 mM l^−1^. Germination seeds of *Satureja hortensis* were counted twice a day for 14 days at laboratory. Then seedling transferred to the greenhouse and their growth continued. Traits measured were seedling weight, seedling lengths, germination rates, germination averages, germination potentials, and percentages of germination. Results showed that the silver nanoparticles improved significantly germination average; plants shoot length and increased plants resistance to salinity. Results showed that a significant reduction in germination percent and seedling growth due to the salinity stress while significantly increased with nano-particles application. In control treatment (without silver nanoparticle) and low level of salinity (0 mM l^−1^) increased seed germination percentage, while the high levels of salinity inhibited the seed germination significantly. The results showed that the effect of silver nanoparticles was significant on germination percentage in *P* ≤ 0.05. Overall, application of silver nanoparticles was beneficial in improving salinity tolerance in the *S. hortensis* seedling and its application may stimulate the differences defense mechanisms of plants against salt toxicity.

## 1 Introduction

Soil salinity is the most significant cause of abiotic stress that plants face, and it limits plant production throughout the world (Tarzi and Fahimi, 2005). Most plant species are sensitive to salt (Jajarmi, 2012). Exposure of plants to diverse kinds of abiotic stresses like drought, salinity, temperature waterlogging, pollution and others is common in nature and occurrence of these stresses has increased in the world today. These stress factors cause a huge economic loss due to their adverse effects on crop productivity and plant growth. Therefore, there is a continuous need to develop new approaches to mitigate the harmful effects of these stresses on plants. In the recent years, nanotechnology is gaining interest of researchers in different fields. Nanoparticles have extremely small size due to which they have acquired some special characteristics, which make them different from their bulk counterparts. Nanoparticles have more solubility, surface area and reactivity as compared to the bulk material. Therefore, they have gained promising position to ameliorate the harmful effects of abiotic and biotic stress to achieve the goal of sustainable agriculture globally. Because of their impact on stress tolerance and nutritional quality of crops, the research related to the application of nanoparticles is increasing. Different types of nanoparticles have been examined for their potential role in protection from biotic and abiotic stresses. These nanoparticles were reported to overcome nutritional deficiencies, to increase enzymatic activities and help in adhesion of plant growth promoting bacteria to plant roots under abiotic stresses and in these diverse ways, improved the tolerance of crops to stresses. These initial reports were quite promising and have opened a new area of utilizing these nanoparticles for increasing crop productivity under stressful environmental conditions. However, in doing so the negative impacts of nanoparticles on environment and plants should not be neglected (Iqbal et al., 2020). Due to the human activities, nanomaterials have been contaminated the environment. These nanomaterials have been gained much more attention from scientists worldwide. The fate and transformation of nanomaterials in agroecosystems were and still one of the most issues all over the world. Therefore, enormous studies have been published concerning these nanomaterials and their applications in different fields including agricultural, medicinal and industrial sectors. The agricultural applications include soil and water nanoremediation, plant nano-nutrition, plant nano-protection, etc. Moreover, the agri-nanotechnology has many environmental and agricultural challenges including agri-sustainability, management of plant diseases and crop protection, remediating the environmental pollution, water management, minimizing the loss of nutrients and their optimizing as well as ameliorating plant abiotic stresses. On the other hand, nanomaterials under certain concentrations may generate and exhibit many toxic effects on plants due to inducing different reactive species like oxygen and nitrogen. Therefore, further studies were need at different levels including molecular and subcellular levels in order to determine the behavior of nanomaterials in inhibiting and/or in inducing plant stress. The mode of action of this behavior also is needed more elucidations under different agroecosystem conditions (Elsakhawy et al., 2018). Salinity significantly increased osmolality, chloride, sodium, and potassium levels of plasma in the fish exposed to AgNPs. The stability of AgNPs in aquatic environments could be regulated by changing the salinity, noting that AgNPs are more stable in low salinity waters (Banan et al., 2020). High salinity can have deleterious effects on an entire plant (resulting in its death) or can result in the loss of a product (Sagghatol-Islami, 2010). Scientists have been trying to encourage plant germination in field conditions because the production of new varieties of transgenic plants and the management of crops with improved germination have gained prominence. One strategy that encourages plant germination in field conditions is the priming of a seed before planting it (Salami et al., 2007). Different priming techniques were now used commercially in many parts of the world.

Recently many studies have showed the physiological responses of plant seedlings to nanoparticles during germination, but the influence of seed germination and root growth varied significantly among the plants and nanopariticles. Nanoparticles could improve fennel seed germination (Feizi et al., 2013). These small size nanoparticles can modify the physiochemical properties of the materials, which can lead to adverse biological effect on living cells (Nel et al., 2006). Many studies have been reported on positive and negative effects of nanoparticles on higher plants. Due to its variable shape and size, it is difficult to predict the positive or negative effect and its mode of action in the environment and within living systems (Holsapple et al., 2005). Silver nanoparticles are one of the most widely used engineered nanoparticles in consumer products where they were increasingly used for their antimicrobial properties (Blaser et al., 2008).

*Satureja hortensis* L., is a medicinal plant endemic to Iran, which is well-known in the folk medicine for its therapeutic uses as herbal tea and as an analgesic and antiseptic substance due to the presence of secondary metabolites including terpenoids, phenolics, flavonoids, steroids and tannins (Hosainzadegan and Delfan, 2009). During recent years, antibacterial, antioxidant, antifungal, antidiabetic, antinociceptive, antihyperlipidemic, antibiofilm, antiinflammatory, antispasmodic and antidiarrhea effects and as well as triglyceride-lowering potential have been reported for *S. hortensis* (Hosainzadegan and Delfan, 2009). In traditional medicine, *Satureja hortensis* L. is used to treat chronic diseases, such as colic, nausea, muscle pain, indigestion, diarrhea, and infectious diseases (Kochaki et al., 2014). Priming techniques were used to affect the plant's metabolic, enzymatic, and biochemical processes and the health of its seeds. Priming techniques improve biological functions and increase seed germination and seedling emergence probabilities. Priming *Satureja hortensis* L., was stimulated a positive physiological and biochemical effect in its seeds before planting in a seedbed (Jajarmi, 2012). Darvishzadeh et al. (2015) found that if *Ocimum basilicum* L. is primed to have improved seed germination under stressful conditions, it is possible to increase the early strength of its seeds, increasing the percentage and rate of its seed germination and increasing its yield. Therefore, this study used a priming technology to increase the plant's resistance to environmental stresses and to evaluate silver nanoparticle on seed priming's effect on germination and the plant's early growth of *Satureja hortensis* L. during in vitro and in vivo conditions.

## 2 Materials and methods

**Figure 1:**
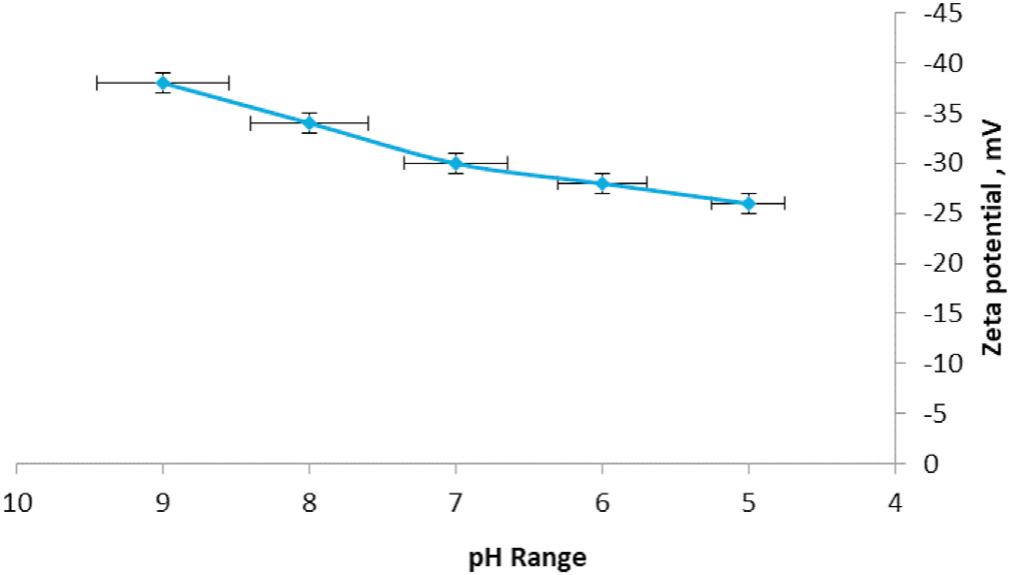
Zeta potential of spherical silver nanoparticles. Zeta potential (mV) values were measured at different pH ranges.

**Figure 2:**
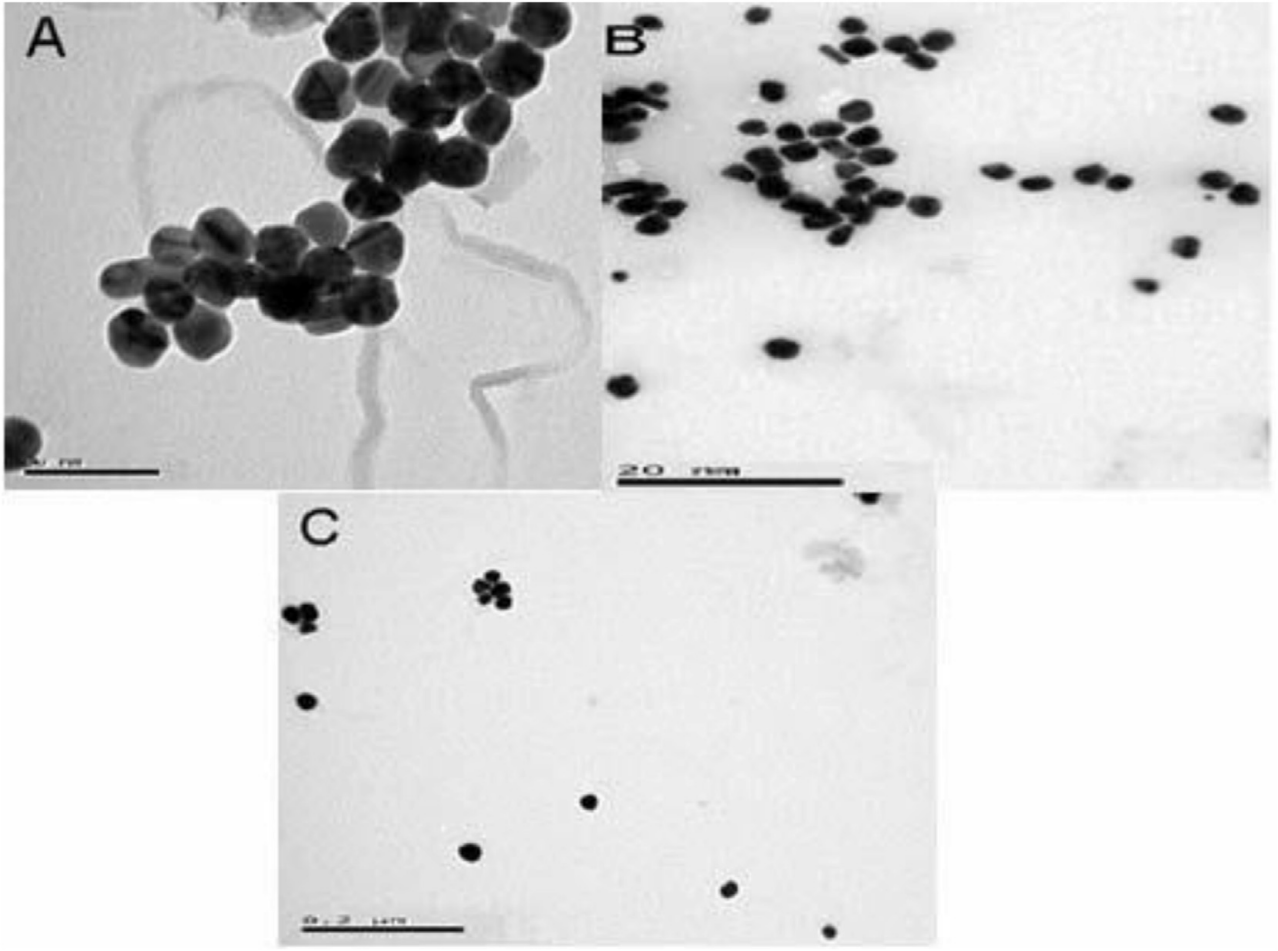
TEM images of spherical Silver, The scale bars were 50nm (A) and magnifications 80 kx, the scale bars were 20 nm (B) and magnifications 100 kx and the scale bars were 200 nm (C) and magnifications 50 kx.

This study was conducted in the laboratory and greenhouse of Department of Agronomy and Plant Breeding in Islamic Azad University of khoy, Iran. It evaluated silver nanoparticles' effect on *Satureja hortensis* L. salt tolerance during the germination stage from 2015 with factorial experiment using a completely randomized design with two factors and three replications. The first factor was silver nanoparticles; a control and 40, 80 and 120 ppm were used. The second factor was salinity levels; distilled water was used as a control, and 30, 60, 90 and 120 mM l^−1^ sodium chloride were used. After the particles were treated to various levels of salinity, a germination-counting phase was conducted. During the counting phase, the number of germinated seeds (those with 2 mm of root growth) was calculated. Nanoparticles as simple molecules, they are in fact complex mixtures. Even in the simplest cases, one must consider the interactions of at least two different aspects of the material. Size domains of nanoparticle is 1–100 nm (Christian et al., 2008). AgNO3 (99.80%) were purchased from Merck. All aqueous solutions were prepared using double distilled water. Dispersions of nanoparticle were sonicated for 20 min and diluted to final concentration of 75 μg/mL in phosphate buffer saline (PBS) pH = 7.4. The zeta potential of pure nanoparticle were measured using a Zetasizer Nano ZS instrument (Malvern, Worcestershire, UK). Zeta values were measured and found to fall between −25.5 and −38.3 mV. These values provide full stabilization of the nanoparticles at different pH, which may be the main reason in producing particle sizes with a narrow size distribution index (Figure 1). The size and morphology of the silver was studied via transmission electron microscopy (TEM) (Jeol, Japan). The microscope was operating at an accelerating voltage of 80 kV. The silver samples were first diluted (1:10) in distilled water, and an aliquot (20 μL) was applied onto a carbon coated grid. The solution was then left for 1 min, and the excess was removed from the grid by blotting with a filter paper. The grids were placed in the grid box for two hours to dry before imaging (Figure 2).

*Satureja hortensis* seed were washed and dried in shade during a week. A solution containg 100 ml of AgNO3 (0.01 M) and *Satureja hortensis* seeds was mixed at room temperature (25 °C) for 48 h with vigorous stirring. Seeds were sterilized in 5% (W/V) sodium hypochlorite (15 min) and washed five times with sterile distilled water. Seeds germinated in pots containing seeds in a growth chamber at 24 (±1) ºC temperature and at a relative humidity of 70%.Germinated seeds were transferred to pots in growth chamber with 17 h light periods and 200 μmol quanta m^−2^ s^−1^ light intensity, day/night temperatures of 25(±1)/18(±1) ºC and irrigated with Hoagland's solution. Thirty-eight days old, plants transplanted into the saline nutrient solutions containing 30, 60, 90 and 120 mM l^−1^ sodium chloride, at pH 6.5, and the nutrient solution was renewed every week. The plants were grown under controlled environment (17 h light periods, 200 μmol quanta m^−2^ s^−1^ light intensity, day/night temperatures of 25(±1)/18(±1) ºC) in a greenhouse. The germinated seeds were counted every alternate day until 30 days had elapsed. When the radicle emerged (≥2 mm), a seed was referred to as having germinated. For each physiological analysis from each treatment, four plants were harvested. Traits measured were root and shoot lengths, dry root and shoot weights, germination rates, mean germination times, and percentages of germination of nanoparticle treatment. Germination rate of nanoparticle treatment can be defined as inverse of time to 50% germination (Bewley et al., 2013). Mean time to germination (MGT) is a measure of the rate and time-spread of germination and percentage germination of nanoparticle treatment is equal to time to 30% of total seed population of seeds with 60% germination and to 40% for a population with 80% germination (Bewley et al., 2013). Then greenhouse phase was conducted. Plants can be grown in a variety of environmental settings including growth rooms, window ledges, outdoors, growth chambers and greenhouses. Peat moss-based mixes, commercial greenhouse mixes, relatively inert media watered with nutrient solutions, and defined agar media can all be employed as plant substrates. Our focus will be on growth of plants on agar and soil in growth chambers and greenhouses. The plant and seed management methods are discussed in the chronological order in would normally be utilized. At the end of this phase, traits measured, were plant height, fresh weights, dry weights, root lengths, and stem lengths.

### 2.1 Statistical analysis

Analysis of variance of the data was carried out using MSTAT-C software. Duncan test was applied to compare means of each trait at p ≤ 0.05.

## 3 Results and discussion

The analysis variance showed that effect of silver nanoparticles on the salinity tolerances of the plants' morphological germination traits under laboratory conditions in Table 1. The silver nanoparticles had significant effect on the stem lengths with a 1% level (Table 1). The highest stem length was observed in the plants treated with a concentration of 80 ppm of silver nanoparticles, and the lowest stem length was observed with the control (Table 2). Ekhtiyari and Moraghebi (2012) compared the effects of silver nanoparticle on the salinity tolerances of the *Cuminum cyminum* L., under laboratory conditions. Their results showed that because penetrate silver nanoparticles into the seed concentration of 40 ppm increased stem length more than other treatments. Safai (2004) revealed that high concentration of sodium chloride and calcium significantly reduced the stem lengths. Such findings are consistent with the present experiment's results. The effect that salinity had significant on stem length (Table 1). The longest stem length was observed in non-saline conditions, and the shortest stem was observed in a saline condition with a salinity of 120 mM l^−1^. Javadi et al. (2014) stated that the reduction of root and stem length in sodium chloride solution is probably due to the toxicity of ions and its negative effect on the cell membrane. Salami et al. (2007) studied the effect of salinity levels of 0, 50, 150, 200 and 250 mM sodium chloride on the morphological characteristics of *Cuminum cyminum* L. Their results showed that salinity reduced root and shoot lengths. Hosseini and Rezvani Moghaddam (2006) showed that salinity can reduce root or stem length and finally decrease seedlings length in Psyllium herb. Silver nanoparticles had significant effect on the Stem weight (Table 1). The highest stem weight was observed in non-salinity and the lowest stem weight was observed with concentration of 120 mM l^−1^ (Table 2). Yazdani Biouki et al. (2020) showed that stem weight of *Callendulla officinalis* decreased with increasing salinity.

**Table 1:** Analysis of variance of nano silver effects and salinity stress on different traits of *Satureja hortensis* L.

Sources of changes				Mean of squares (MS)				
	df	Stem length	Stem weight	Root length	Root weight	Mean germination time	Germination rate	Germination percentage
Silver Nano	3	455.24∗∗	7.46	164.86∗∗	7.54	5.64∗∗	7.62∗∗	1028.8∗∗
Salinity	4	6368.6∗∗	9.71∗	69.25∗∗	54.6∗∗	4.07∗∗	5.12∗∗	559.9∗∗
✕ Salinity Silver Nano	12	123.9	5.97	37.68	16.6	0.88	1.48∗∗	109.41∗∗
Error	22	71.26	3.1	23.95	9.3	0.45	0.3	20.2
C.V		11.39	13.37	21.12	28.45	11.8	8.65	6.23

∗∗,∗ significant difference at the probability level of 5% and 1% respectively.

**Table 2:** Comparing the mean of different treatments of nano silver and salinity on measured traits *Satureja hortensis* L.

Treatment		Stem length (mm)	Stem weight (mg)	Root length (mm)	Root weight	Mean germination time
Silver Nano (ppm)	0	67.13 c	14.90 a	18.67 c	5.6 a	6.42 a
	40	73.27 b	14.91 a	23.73 a	5.7 a	5.71 b
	60	81.73 a	14.92 a	25.8 a	5.8 a	5.03 c
	80	74.4 b	14.91 a	24.53 a	5.7 a	5.32 bc
Salinity (mM l^−1^)	0	105.1 a	14.92 a	30.67 a	5.8 a	4.7 c
	30	83.92 b	14 ab	25.58 b	4.8 b	5.46 b
	60	77.58 b	12.73 bc	21.75 bc	4.7 b	5.72 ab
	90	58.42 c	11.78 cd	20.92 cd	4.01 c	5.97 ab
	120	45.67 d	10.82 d	17 d	3.8 d	6.22 a

The highest root length was observed in the treatment of nano silver, all treatment was in a same statistical group. The lowest root length was observed in control treatment. Darvishzadeh et al. (2015) also reported that silver nanoparticle stimulus on germination rate and increased amount of plant growth regulators in seeds. The highest root length was observed in control (the absence of salinity) and the lowest root length was observed in a concentration of 120 mM l^−1^ (Table 2). Some studies have shown that germinated seeds have shorter roots in saline environments and sodium chloride has a more severe inhibitory effect on the appearance of embryonic tissues (Khot et al., 2012).

The effect of salinity on root weight was significant (Table 1). The highest root weights were observed in salt insufficiency and the lowest root weight in salinity conditions with a concentration of 120 mM l^−1^ (Table 2). Since rooting length was higher in distilled water and more growth, the highest root weight was observed in this treatment. Reducing the osmotic potential and the effects of ionic toxicity, with increasing salinity levels, interfere with the root growth process, which leads to a reduction in root dry weight (Iqbal et al., 2020). The effect of silver nanoparticles had significant effect on mean germination time at 1% level (Table 1). The highest germination time was observed in control or non-use of nano silver and the lowest germination time was observed in plants treated with concentration of 80 ppm (Table 2). Nanoparticles have been effective at the rate of germination and increase plant growth, which is due to penetrated these particles into seeds (Khataee and Mansoori, 2011). Khodakovskaya et al. (2011) reported that penetrating nanoparticles in tomato seeds increased germination by increasing the absorption capacity of water by seeds. Darvishzadeh et al. (2015) also reported the positive effects of silver nano particles on the rate of germination. The lowest average germination time was observed in control and the maximum germination time was observed with concentration of 120 mM l^−1^. In unfavorable environment, germination is delayed and the average germination time is increased, which is not desirable (Jajarmi, 2012). Some researchers believe that at salinity conditions with increasing the osmotic pressure and decreasing water absorption by seeds, as well as by the toxic effects of sodium and chloride ions increased germination time and delayed germination seeds (Soltani et al., 2006).

**Figure 3:**
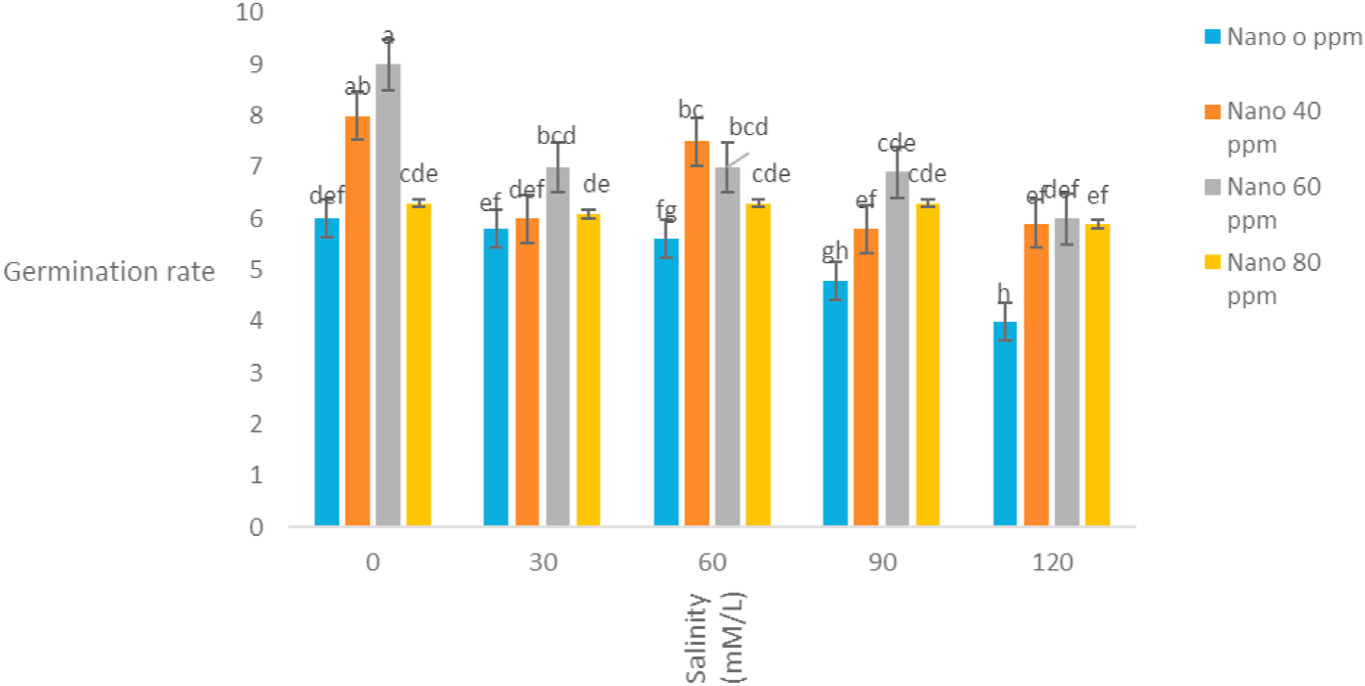
Interaction of salinity and nano silver on Germination rate of *Satureja hortensis* L.

**Figure 4:**
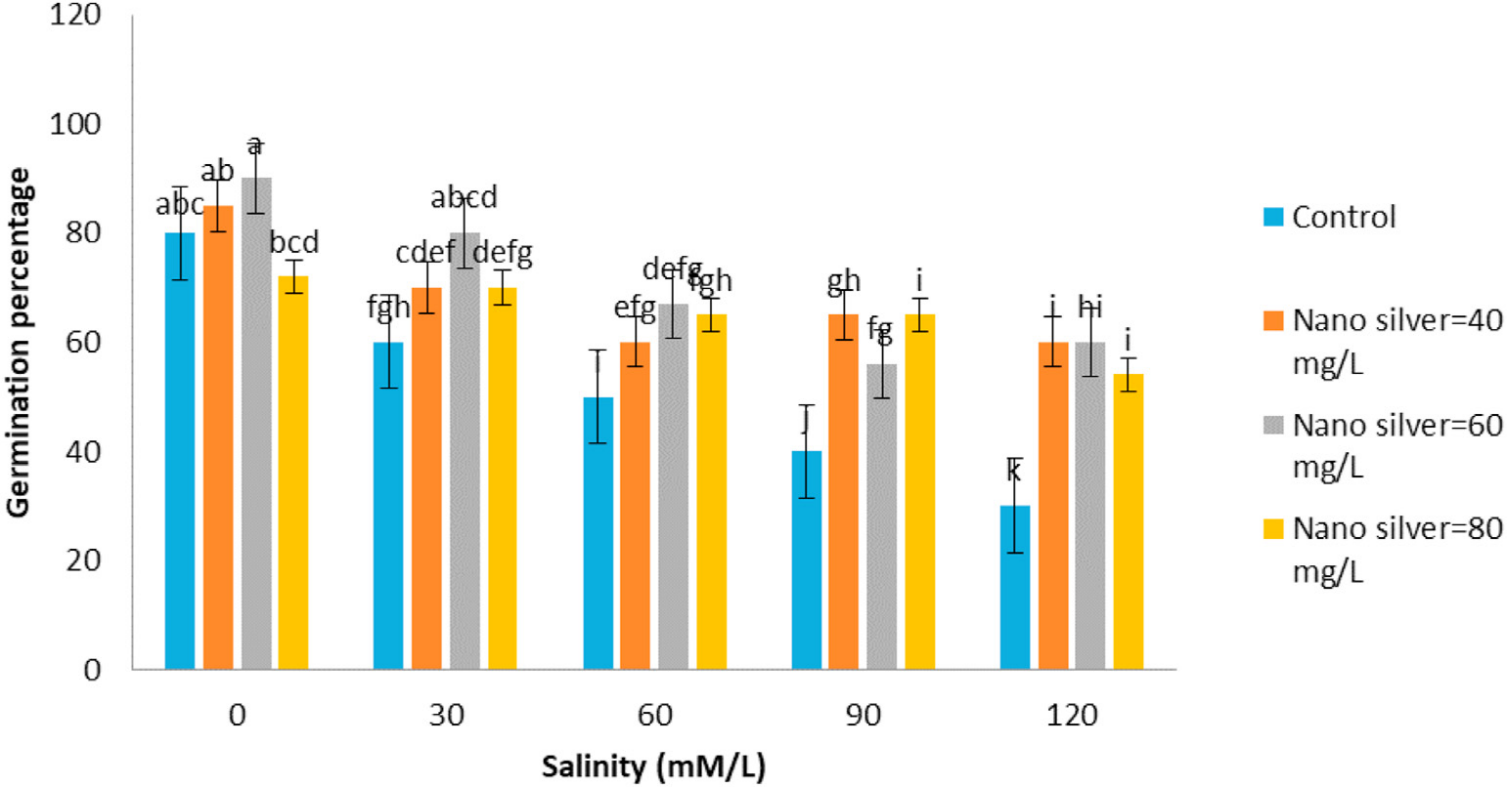
Interaction of salinity and nano silver on Germination percentage of *Satureja hortensis* L.

The simple effects of salinity and nano silver and their interaction on germination rate were significant at 1% level (Table 1). The highest rate of germination was observed in 80 ppm nano silver with insufficient salinity and the lowest germination rate was observed in the non-application of nano silver with a salinity of 120 mM l^−1^ (Figure 3). Nanoparticles are effective in increasing germination speed, because in the absence of salinity, it also increases the rate of germination, and in the salinity condition, nano silver is effective in reducing the adverse effects of salinity, and the effect of nano silver is increased with increasing salinity. Safai (2004) stated that with increasing salinity, the rate and percentage of fennel germination decreased. The highest germination percentage was 80 ppm in nano silver application, with insufficient salinity and the lowest germination percentage was also observed in 120 mM l^−1^ salinity and non-application of nano silver (Figure 4). According to the results, *S. hortensis* is susceptible to salinity under laboratory conditions and, with increasing salinity, its germination percentage is reduced, and also nano silver is effective to increasing germination percentage, because in the absence of salinity, the percentage of germination is increased. In salinity conditions, nanosilver has been effective in reducing the adverse effects of salinity and, with increasing salinity, the effect of nano silver has been increased. Ekhtiyari et al. (2011) also stated that the treatment of 20 ppm nanosilver particles had significant effect on germination indices of fennel plants and increased their resistance to salinity. Islam and Karim (2010) reported that, seedling length, germination percentage, germination rate and seedling germination index of three species of medicinal plants of Sardinia, Chicory and Artemisia significantly decreased at salinity conditions. Ekhtiyari and Moraghebi (2012) studied the effects of nanosilver particles on crop salinity tolerance in germination stages and observed that treatment of 20 ppm nanosilver has significant effect and increases the resistance to salinity of the cumin plant.

**Figure 5:**
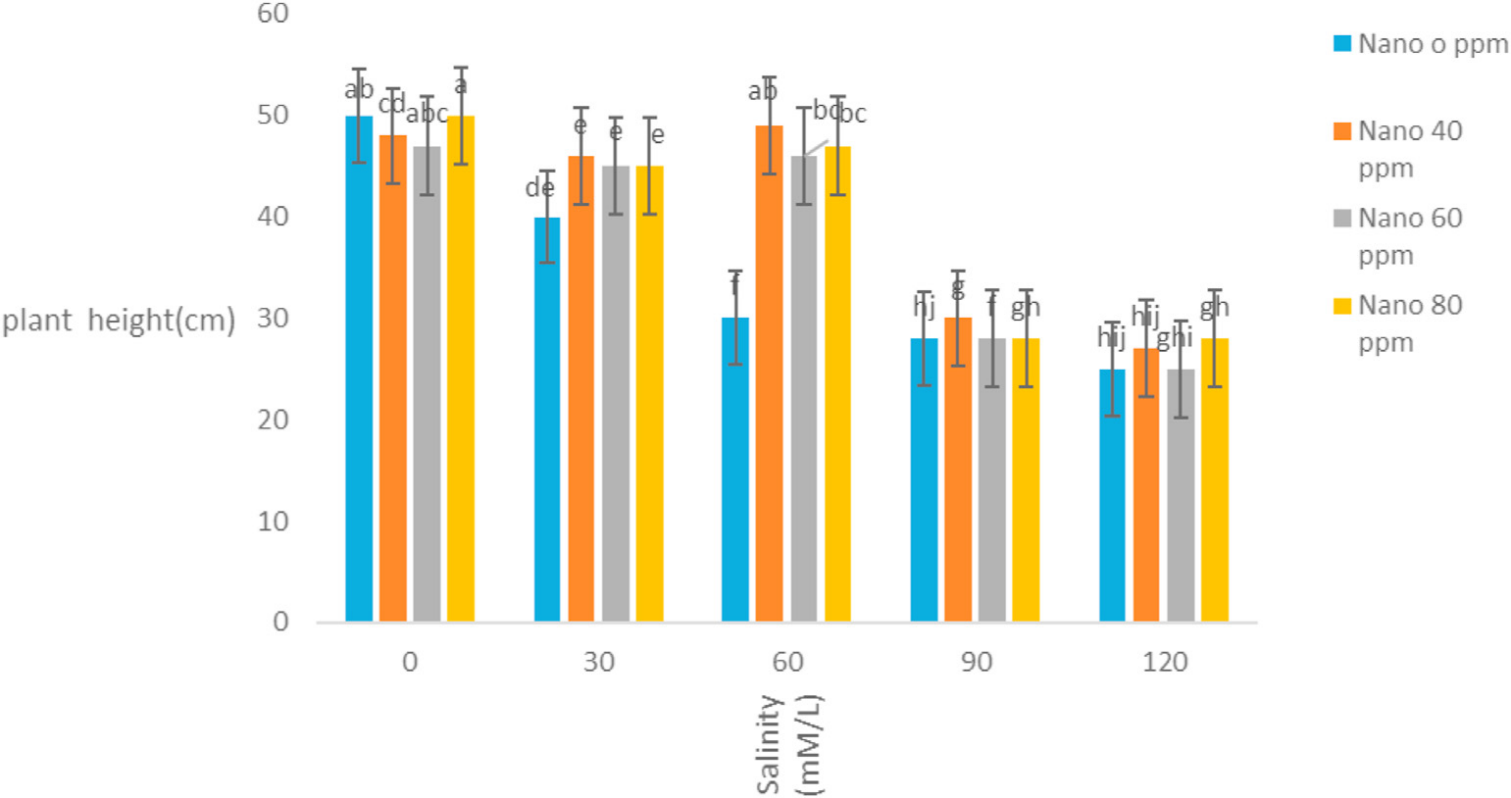
Interaction of salinity and nano silver on Plant height of *Satureja hortensis* L.

**Figure 6:**
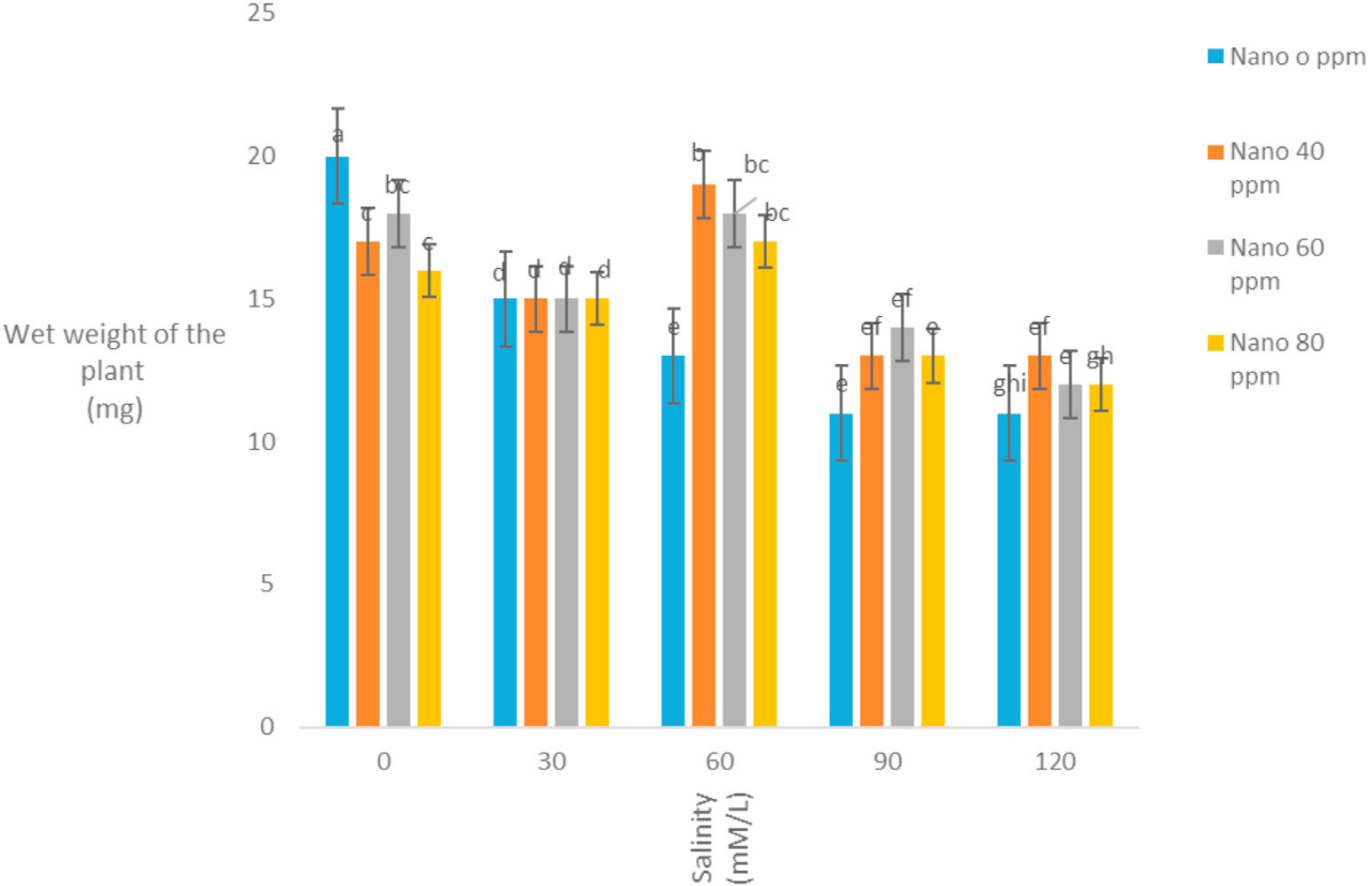
Interaction of salinity and nano silver on Wet weight of the plant of *Satureja hortensis* L.

**Figure 7:**
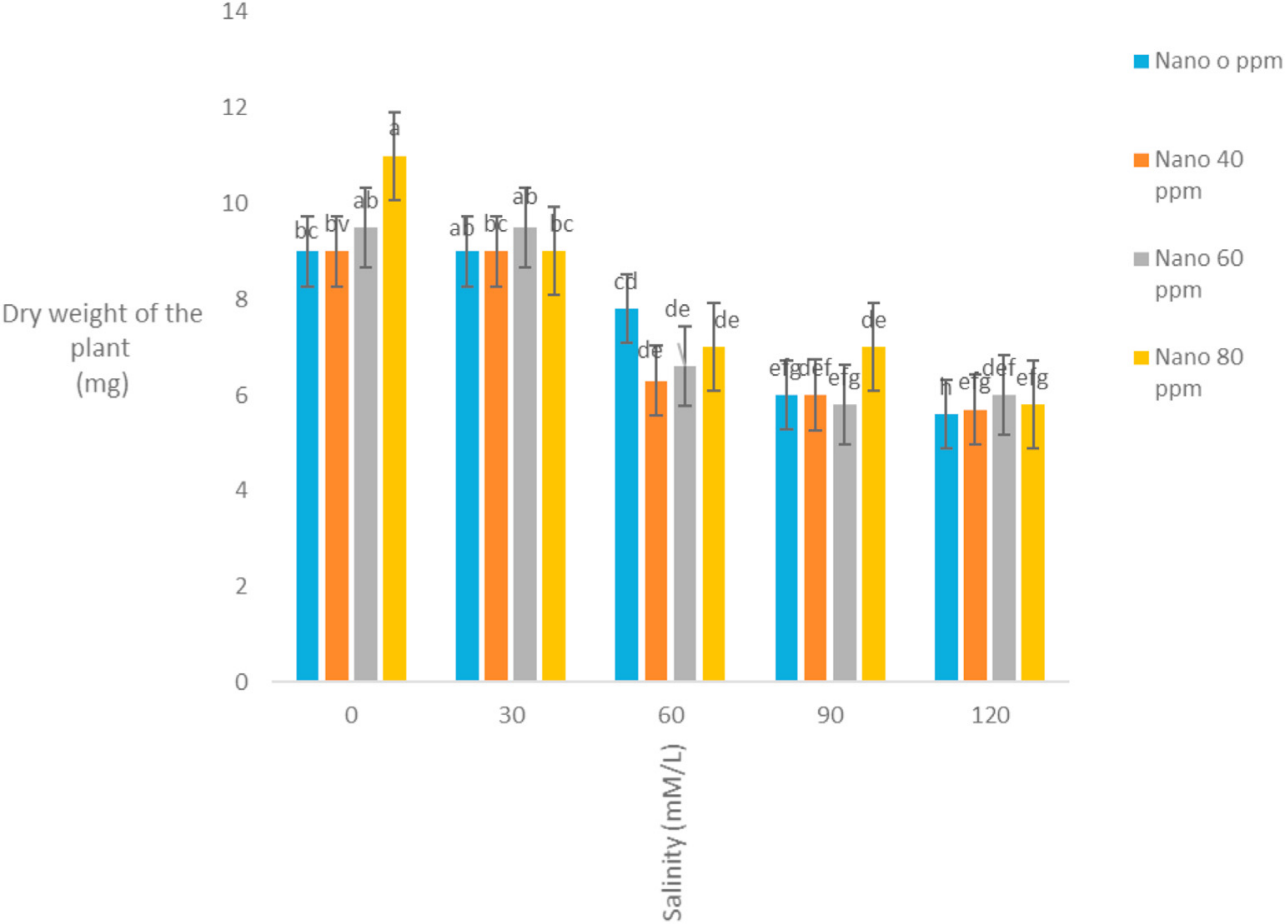
Interaction of salinity and nano silver on dry weight of *Satureja hortensis* L.

**Figure 8:**
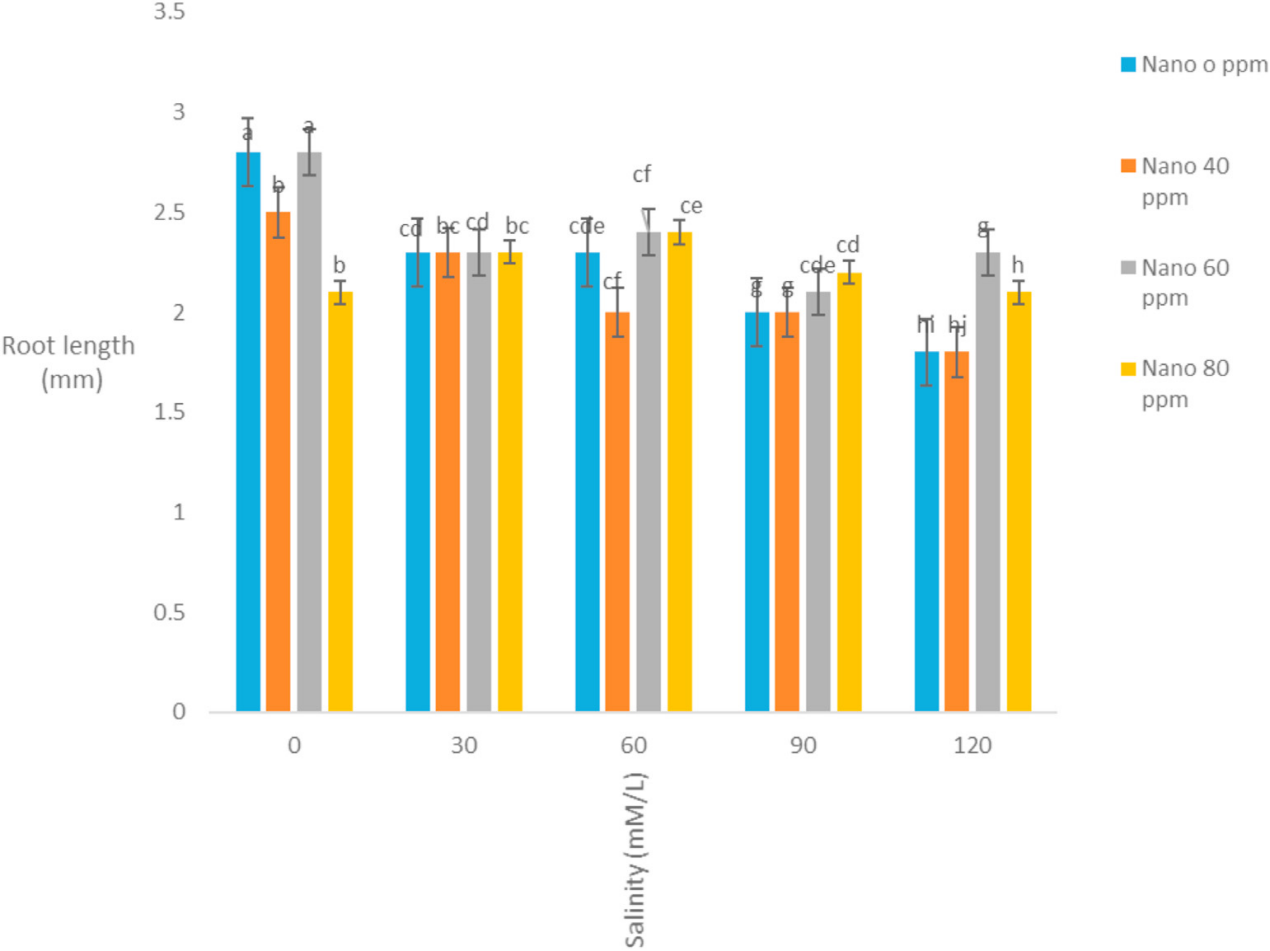
Interaction of salinity and nano silver on Root length of *Satureja hortensis* L.

**Figure 9:**
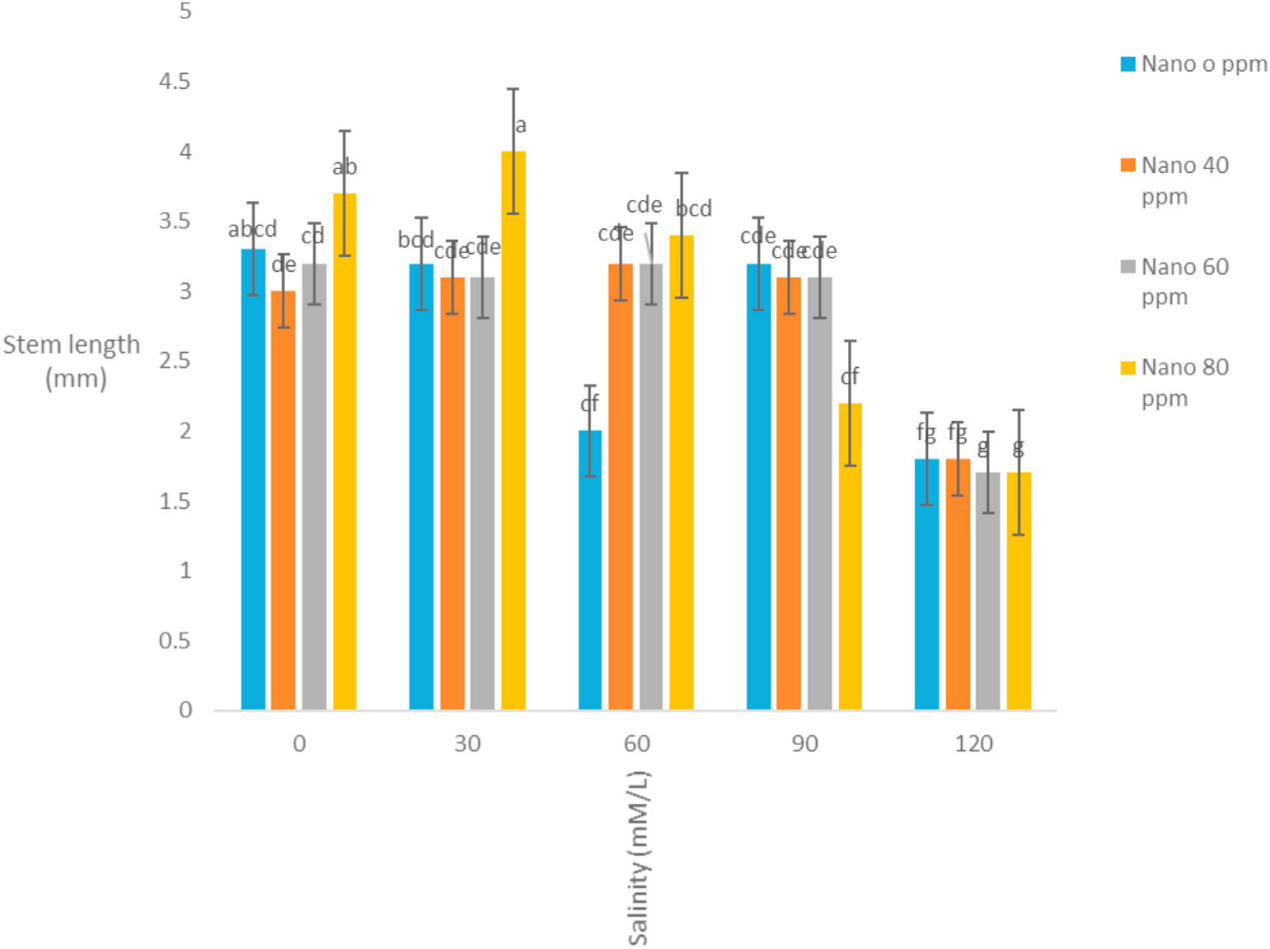
Interaction of salinity and nano silver on Stem length of *Satureja hortensis* L.

**Figure 10:**
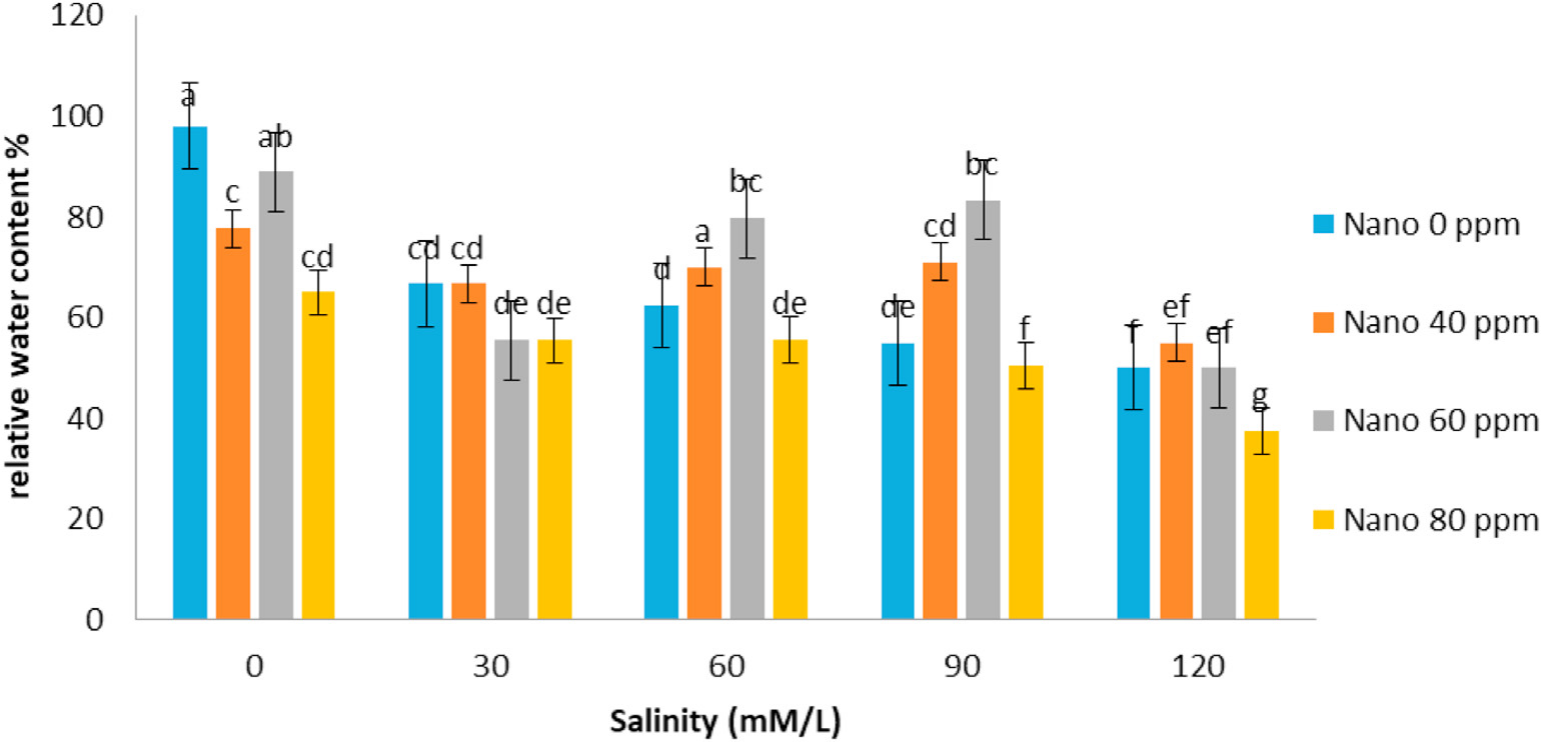
Interaction of salinity and nano silver on relative water content of *Satureja hortensis* L.

The correlation coefficients of traits showed that stem length had a positive correlation with all traits except the mean germination time. The highest correlation coefficient between stem length and stem weight (r = 0.92) was observed (Table 3). It seems that with increasing stem length, the growth of the stem increases and the stem weight rises. Evaluation of correlation coefficients of traits showed that root length was positively correlated with all traits except the mean germination time. The highest correlation coefficient between root length and root weight (r = 0.87) was observed (Table 3). It seems that with increasing root length, the growth of the root increases and the root weight rises. Root length also had high correlation coefficient with germination rate. Evaluation of correlation coefficients of traits showed that germination percentage had a positive correlation with all traits except the mean germination time. The highest correlation coefficient of germination percentage with germination rate (r = 0.87) was observed (Table 3). Babaia et al. (2011) also stated that there is a high correlation between germination percentage and germination rate. Evaluation of correlation coefficients of traits showed that the mean germination time with all traits was negative and the highest negative correlation coefficient was observed with germination rate and germination percentage (Table 3). Iran nezhad and Shahbazian (2004) and Jajarmi (2012) stated that the cultivars with a mean germination time had a higher rate and germination percentage, which is especially effective in lowering the humidity during plant deployment. The analysis variance of the effect of silver nanoparticles on the salinity tolerances of the plants' morphological germination traits under greenhouse conditions showed that in Table 4. Table 4 showed that the plant height decreased with increasing salinity concentration of 120 mM l^−1^ as compared to control. Seeds treated with silver nanoparticles at 60 ppm concentration had better performance (Figure 5). Moghadam et al. (2015) reported the effect of salinity stress on some functional parameters and morphological characteristics of green mint in hydroponic conditions. Their results concluded that salinity had a significant effect on leaf area traits, leaf fresh weight, leaf dry weight, stem, root, plant height, number of stems, stem length, and number of nodes. The highest plant height was observed in control treatment and the lowest plant height was observed in treatments 90 and 120 mM l^−1^ salinity. Archangi et al. (2012) in studying the effect of salinity stress on morphological characteristics and the amount of sodium, potassium and calcium in the *Trigonella foenum graecum* L. under hydroponic conditions reported that with increasing salinity concentration reduced plant height, shoot dry weight, root dry weight and leaf number. Fresh weight of this plant in different treatments had been shown in Table 4. The highest fresh weight in the control treatment was obtained with a mean of 63.62 (Figure 6). Moghadam et al. (2015) concluded that, salinity had a significant effect on fresh weight, leaf dry weight, leaf area, stem, root, plant height. The highest fresh weight was observed in control treatment and the lowest fresh weight was observed in 90 and 120 mM l^−1^ salinity levels. Salinity had no significant effect on shoot fresh weight to ratio root, fresh leaf to root weight, leaf weight, number of stem and stem diameter. According to the results, *S. hortensis* seems to be sensitive to salinity and cannot withstand the salinity of more than 30 mM l^−1^ sodium chloride. The results showed that the dry weight of the plant decreased with increasing salinity concentration, so that control treatment with 10.88g has the maximum value and the minimum dry weight (4.79) obtained in concentration 120 mM l^−1^ salinity (Figure 7). Silver nanoparticles increased the root length to 2.76 mm in control plant (Figure 8). The highest root length was obtained in control treatments with 30 mM l^−1^ salinity and the lowest root length was obtained with 120 mM l^−1^ salinity. Salinity in Basil decreased root length (Sharafi 2008). Reduction of growth parameters (stem diameter, intercalation interval, inflorescence length, number of nodes) was observed in mint under salinity stress even in low sodium chloride treatments (Javadi et al., 2014). The silver nanoparticles with 80 ppm concentration had good effect on the stem length of the *S. hortensis* compared to its other concentrations. The maximum stem length was observed at a concentration of 30 mM l^−1^, salinity and 80 ppm of silver nanoparticle concentration (Figure 9). Because of plants with different ages were used this difference has been seen. Lowering height in *S. hortensis* was obtained from reduced stem length under salinity stress. In salinity levels greater than 30 mM l^−1^, burns, leaf necrosis and their loss were observed. The highest relative water content in the control treatment was obtained and the least relative water content was observed at a concentration of 120 mM l^−1^, salinity and 80 ppm of silver nanoparticle concentration (Figure 10). Seed germination loss at high concentrations of salinity in the present study can be due to a decrease in the water content of the seed and its effect on physiological processes such as transpiration, respiration and photosynthesis. In the present results, a reduction in RWC with the use of salinity especially at a concentration of 120 mM l^−1^ was obtained. These symptoms and the reduction of the above characteristics because increased water content can explain revealed effects and indicate that, this plant was salt-sensitive and cannot withstand more than 30 mM l^−1^ salinity. Increasing the salinity level in the dark environment of the Lamiaceae family reduces the growth and leads to a reduction in stem length and body weight. Reduction of shoot length and shoot weight in green peppermint in salinity stress due to reduction of plant photosynthesis (Babaia et al., 2011). Since salinity is one of the increasing problems in the world, it also covers a large part of our country (Archangi et al., 2012), investigating and developing physiological techniques such as germination, to survive in a variety of stresses, especially salinity stress, for increasing the quality parameters of seeds, including, is essential as one of the most important environmental stresses. The negative effects of salinity are evident due to osmotic pressure, ion toxicity, and collapse in plant nutrition balance (Demir kaya, 2009). Archangi et al. (2012); Demir kaya (2009) reported in many studies that increased salinity constituted a decrease in the seed germination rate, as well as salinity delayed the germination process and prolonged the germination process by reducing osmotic potential, ionic toxicity, and imbalance in food intake (Demir kaya, 2009). Desirable germination and rapid seedling growth will result in better deployment and reduced competition with other plants. Increasing salinity levels reduced seed germination and seedling growth. Effect of different concentrations of sodium chloride and nano silver particles to improved germination percentage of basil seeds (Darvishzadeh et al., 2015) showed that the seeds treated with 40 ppm of nano silver exhibited more tolerance than other treatments even at different salinity levels. The results of salinity stress showed that high concentrations of sodium chloride and calcium could provide an inappropriate environment for germination of seeds. It can be observed that with increasing salinity, the percentage and rate of germination in all treatments of nano silver under laboratory conditions (germinator) has decreased and is consistent with the reports of other researchers. Sharafi (2008), Tarzi and Fahimi (2005), Demir kaya (2009), Davazde Emami (2002) showed that high concentrations were observed in the effects of salinity stress in *Callendulla officinalis*, *Cuminum cyminum*, *Helianthus annuus*, and *Foeniculum vulgare*. Sodium chloride and calcium significantly reduced the percentage and rate of germination, which is in agreement with the results of the present experiment. Davazde Emami (2002) showed that, with increasing salinity, the percentage and speed of germination of seedlings of medicinal plants, as in other agricultural products, decreased. Tarzi and Fahimi (2005) reported that the germination of *Cuminum cyminum* decreased with increasing salinity levels, and stated that most of the plants in the germination stage are more susceptible to salinity than other growth stages, although there are some exceptions for the *Cuminum cyminum* species in the bud stages to Salinity has a relative resistance. Soltani et al. (2006) By applying salinity stress on *Cuminum cyminum*, reported that salinity stress had significant effect on germination uniformity, cumulative percentage of germination, germination rate, seedling length and root to shoot ratio at a probability level of 1%. Silver nanoparticles treatment increased the rate of germination compared to the control treatment. This trend is very evident in Sardari, which is consistent with the findings of Ekhtiyari and Moraghebi (2012) on the effect of silver nanoparticles treatment on increasing germination percentage of *Cuminum cyminum*, compared to control treatment.

**Table 3:** Correlation coefficients between different traits *Satureja hortensis* L.

Traits	Stem length	Stem weight	Root length	Root weight	Germination percentage	Germination rate
Stem length	1					
Stem weight	0.92∗∗	1				
Root length	0.63∗∗	0.52∗∗	1			
Root weight	0.26∗	0.24∗	0.89∗∗	1		
Germination percentage	0.49∗∗	0.43	0.72∗∗	0.69∗∗	1	
Germination rate	0.52∗∗	0.46∗∗	0.81∗∗	0.78∗∗	0.87∗∗	1
Average germination time	-0.476∗∗	-0.25	-0.31∗	-0.21	-0.72∗∗	-0.78∗∗

∗∗,∗ significant difference at the probability level of 5% and 1% respectively.

**Table 4:** Analysis of variance of nano silver effects and salinity stress on different seedling traits *Satureja hortensis* L.

Sources of changes			Mean of squares (MS)			
	df	Plant height	Stem length	Root length	Fresh weight of the plant	Dry weight Of the plant
Silver Nano	3	19.89∗∗	0.07∗∗	0.31∗∗	57.63∗∗	4.41∗∗
Salinity	4	521.09∗∗	1.66∗∗	3.97∗∗	1032.53∗∗	125.13∗∗
✕ Silver Nano Salinity	12	24.16∗∗	0.10∗∗	0.23∗∗	41.40∗∗	9.34∗∗
Error	22	2.24	0.010	0.016	0.90	0.54
C.V		11.33	3.66	6.99	3.23	12.48

∗∗,∗ significant difference at the probability level of 5% and 1% respectively.

Hojat and Kamvab (2017) showed the great effects of silver nanoparticles to improve salinity stress on Fenugreek seed germination. In without silver nanoparticle low level of salinity (0 dS m^−1^) increased seed germination percentage, while the high levels (5, 10, 15 and 20 dSm^-1^) inhibited the seed germination significantly. The effect of AgNPs was significant on germination percentage and beneficial in improving salinity tolerance in the Fenugreek seedling.

The remarkable drought-resistance of the terrestrial cyanobacterium *Nostoc flagelliforme* (*N. flagelliforme*) has attracted attention for many years. The results shed light on the relationship between carotenoid-binding proteins and the desiccation resistance of terrestrial cyanobacteria, and the physiological functions of carotenoid-binding protein complexes in relation to desiccation are discussed (Yang et al., 2019). Soil salinization represents one of the major limiting factors of future increase crop production through the expansion or maintaining of cultivation area in the future. High salt levels in soils or irrigation water represent major environmental concerns for agriculture in semi-arid and arid zones. Recent advances in research provide great opportunities to develop effective strategies to improve crop salt tolerance and yield in different environments affected by the soil salinity. It was clearly demonstrated that plants employ both the common adaptive responses and the specific reactions to salt stress. The research results presented here may be helpful to understand the physiological, metabolic, developmental and other reactions of crop plants to salinity, resulting in the decrease of biomass production and yield. In addition, the modern studies on how to mitigate salt stress effects on photosynthetic apparatus and productivity of crop plants with the help of phytohormones, glycinebetaine, proline, polyamines, paclobutrazol, trace elements and nanoparticles. To understand well these effects and to discover new ways to improve productivity in salinity stress conditions it is necessary to utilize efficiently possibilities of promising techniques and approaches focused on improvement of photosynthetic traits and photosynthetic capacity, which determines yield under salt stress conditions (Mbarki et al., 2018).

The results indicated that soaking tomato transplants in Silver nanoparticles for 24 h had no significant effect on increasing tomato tolerance to sodium chloride salt within the used levels. This was reflected in negative effects on fruit number per plant, fruit diameter, average fruit weight, number of branches per plant and plant height. The control treatment (soaked in distilled water) of SNP was consistently superior for all studied traits. SNP treatments reduced the fruit number per plant, fruit diameter, average fruit weight, number of branches per plant and plant height (Nabil et al., 2015). Silicon (Si) is reported to reduce the effect of salinity on wheat (*Triticum aestivum* L.) and other crops. It is concluded that Si may have improved shoot growth of the salt-resistant as well as the salt-sensitive wheat genotype by decreasing plant Na^+^ uptake and shoot: root Na^+^ distribution as well as by increasing glutathione concentration. Silicon may have also improved in-plant Na^+^ detoxification by increasing cell-wall Na^+^ binding (Saqib et al., 2008). Although joint efforts by research communities generated essential knowledge of the impacts of AgNPs on plants, most of these experimental outcomes were based on laboratory experiments under controlled conditions that are likely far from field conditions, such as the exposure method (hydroponic vs. soil), exposure dosage, and time (acute vs. chronic). Therefore, it is hard to predict whether the phytotoxicity of AgNPs and tolerance mechanisms under laboratory conditions are the same as under field conditions. To this end, the establishment of well-designed, plant life-cycle experimental systems under environmentally realistic conditions is required to accurately evaluate the impacts of AgNPs on plants and to generate environmentally relevant implications. In addition, most studies performed during the last decade focused on the impacts of AgNPs on plants at the morphological and physiological levels; however, the profound impacts of AgNPs at the molecular level did not draw enough attention. Benefits from the development of systems biology and multiple omics methodologies, such as transcriptomics, proteomics, and metabonomics, can be employed in future studies to comprehensively assess the phytotoxicity mechanism of AgNPs and tolerance mechanisms in plants (Yan and Chen, 2019).

Nanosilver application resulted in enhanced leaf and bulb biomass, leaf greenness index, and flower abundance. The effects of Nanosilver on plant growth and the content of assimilation pigments and some macronutrients depended on nanoparticle concentration. The unique properties of Nanosilver may be highly beneficial in the cultivation of plants, but, as their mechanisms of action are not fully understood, further detailed mycological, biochemical, and molecular studies on the impact of nanosilver on plant health and stress are necessary (Yan and Chen, 2019). Salinity stress is a critical environmental issue that affects crop production globally. Salinity stress can particularly inhibit seed germination and early seedling development of seeded crops like savory. Seed priming or pretreatment with nanoparticles have been shown to promote seed germination of various crops (Mahakham et al., 2016). Increased water content was observed in the nanomaterial treated seeds during germination when compared to the controls (Nair et al., 2012). Therefore, the CNP-derived osmotic adjustment between the plant and the substrate may maintain water uptake and cell turgor for growth. Unlike salt stress, lettuce seed thermoinhibition is mainly due to the de novo synthesis of Abscisic Acid (ABA) induced by high temperature (Huo et al., 2013). Salt stress through enhancement of osmotic pressure leads to the decrease of germination percentage, germination rate, germination index and an increment in mean germination time of *S. hortensis* seeds. For overcoming the negative impacts of salinity on the plant, growth and yield can be to attempt to new strategies. The dry and fresh weight of seedlings diminished as seedling length declined with increasing salinity levels since root number, shoot number, root length and shoot length decreased essentially. Results demonstrate that nanoparticle treatments enhances seed germination, promptness index, and seedling growth. The positive effect of nanoparticle treatments on physiological properties was in conditions that the plant grew under salt stress was more increasingly exceptional in examination with the conditions that plant grown under normal conditions. The results of this study showed that nanoparticle treatments could be involved in the metabolic or physiological activity in higher plants exposed to abiotic stresses.

## 4 Conclusions

This finding is consistent with our finding that 80 ppm silver nanoparticles have improved germination speed, plant height, and stem length compared to control treatment. Despite the gradual increase in salinity levels, silver nanoparticles treatment at 80 ppm concentration did not stop germination at high salinity, which means that silver nanoparticles increased the root and shoot lengths of the *S. hortensis*. Silver nanoparticles improving the competitiveness of the plant and possibility of better use of the plant from the environmental conditions of water and light for photosynthesis is better in salinity conditions. In general, it can be admitted that the application of silver nanoparticles in *S. hortensis* increases the yield of seed germination and growth of the this plant in greenhouse conditions, which makes it possible to easily establish the conditions for survival.

## Declarations

### Author contribution statement

All authors listed have significantly contributed to the development and the writing of this article.

### Funding statement

This work was supported by the Faculty of Agriculture, Department of Horticulture, Khoy Branch, Islamic Azad University, Khoy, Iran.

### Data availability statement

Data included in article/supplementary material/referenced in article.

### Declaration of interests statement

The authors declare no conflict of interest.

### Additional information

No additional information is available for this paper.
